# Double encapsulation of fucoxanthin using porous starch through sequential coating modification with maltodextrin and gum Arabic

**DOI:** 10.1002/fsn3.1411

**Published:** 2020-01-20

**Authors:** Najme Oliyaei, Marzieh Moosavi‐Nasab, Ali Mohammad Tamaddon, Mahboubeh Fazaeli

**Affiliations:** ^1^ Seafood Processing Research Group School of Agriculture Shiraz University Shiraz Iran; ^2^ Department of Food Science and Technology School of Agriculture Shiraz University Shiraz Iran; ^3^ School of Pharmacy and Research Center for Nanotechnology in Drug Delivery Shiraz University of Medical Science Shiraz Iran

**Keywords:** double encapsulation, fucoxanthin, gum Arabic, maltodextrin, porous starch

## Abstract

This study aims to assess the effect of gum Arabic (GA), maltodextrin (MD), or their combination as a coating agent at different ratios (1/3, 1/5, and 1/7 w/w) to encapsulate fucoxanthin. For this purpose, fucoxanthin was initially extracted and purified from *Sargassum angustifolium* brown seaweed and then loaded into porous starch (PS). The fucoxanthin‐loaded PS samples were further contributed in another encapsulation process using the coating materials. All samples were evaluated in terms of encapsulation efficiency, Fourier‐transform infrared (FTIR) spectroscopy and stability under light, dark and low or high temperature (4 and 50°C) exposure over a certain time period. Purification of fucoxanthin was verified through HPLC and NMR spectroscopy. It was shown that the subsequent coating with MD + GA (1/7 w/w) caused an enhanced encapsulation of fucoxanthin‐loaded PS, reaching to about 96%. In addition, the stability of fucoxanthin‐loaded PS was greatly influenced by light and high temperature exposure and decreased from 85% to 58% using the GA‐coated material (1/3 w/w). First‐order kinetic model was found to be fitted well on thermal degradation data of fucoxanthin. Interestingly, the mixture of MD + GA (1/7 w/w) exhibited the highest fucoxanthin prevention at the end of the storage period. Conclusively, the findings of this study can provide simple and facile protocol for food chemists in protecting the food ingredients using encapsulation process.

## Graphical abstract

Fucoxanthin has potential therapeutic benefits for several major diseases. This carotenoid is heat and light sensitive, and thus, encapsulation of fucoxanthin is necessary. Porous starch is a novel carrier for encapsulation of bioactive compounds that can be modified with gum Arabic (GA) and maltodextrin (MD). The modification of encapsulated fucoxanthin‐loaded PS using MD and GA as coating agents has excellent encapsulation efficiency and stability. This encapsulation protocol can be useful for those industries that need to protect their sensitive ingredients from severe environmental conditions.

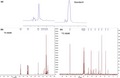

## 1 INTRODUCTION

Carotenoids are liposoluble natural pigments with well‐known biological function and health beneficial effects (Rostamabadi, Falsafi, & Jafari, 2019). Fucoxanthin is one of the major carotenoids available abundantly in various species of brown seaweeds. Several reports have recently given special attention into beneficial health effects of fucoxanthin in preventing cancer, inflammation, diabetic, and obesity (Mok, Yoon, Pan, & Kim, 2016). Despite these effects, fucoxanthin suffers from some limitations such as sensitivity to light, heat, oxygen, and metals, due to the presence of unsaturated structure in its backbone (Quan, Kim, Pan, & Chung, 2013). Recently, to overcome its drawback or to minimize its vulnerability to various environmental conditions, a variety of nanoencapsulation strategies such as nano‐liposomal vehicles, niosomes, cubosomes, hexosomes, and nanoemulsions has been introduced which efficiently insure the safe passage of carotenoids throughout the gastrointestinal (GI) tract and their subsequent sustained release at the intended sites (Rostamabadi et al., 2019). Moreover, numerous fucoxanthin encapsulation methods such as solid lipid particle (Quan et al., 2013), nanoemulsion (Huang et al., 2017), and nanogel (Ravi, Arunkumar, & Baskaran, 2015) have been suggested. One approach to encapsulate the fucoxanthin is to take benefit of cheap biopolymers such as starch.

Starch is known as the main component of cereal grains and has found broad variety of applications in pharmaceutical, food, and nonfood industries. Starch, in its native form, does not always possess the satisfactory physicochemical properties that meet certain types of processing due to its inherent brittleness, high swelling, and weak molecular structure (Singh, Nath, & Singh, 2010). To cope with this issue, numerous physical, chemical, and enzymatic modifications have been performed to improve the functional properties of starch for food or nonfood‐based applications (Patindol, Shih, Ingber, Champagne, & Boue, 2013). Among different types of starch, porous starch (PS) is viewed as a relatively new modification of starch with pore size of about 1 µ m (Zhang et al., 2012). Due to its porous structure, PS can be readily employed as an adsorbent of heavy metal ions (Ma, Liu, Anderson, & Chang, 2015), designing drug delivery systems (Wu et al., 2011) and development of carrier for sensitive additives (Belingheri, Curti, Ferrillo, & Vittadini, 2012). Starch can be modified through various physical (microwave, ultrasound), chemical (solvent exchange), and biological (enzymes) techniques to create honeycomb‐like structure of PS (Belingheri, Ferrillo, & Vittadini, 2015; Oliyaei, Moosavi‐Nasab, Tamaddon, & Fazaeli, 2019). Among these techniques, the solvent exchange is a common approach for the synthesis of PS that can be carried out through sequential steps of (a) gelatinization of starch, (b) solvent exchange with alcohol, and (c) evaporation of alcohol, which eventually leaves several pores in its structure (Cai, Chen, Hong, Liu, & Yuan, 2013).

Along with using starch, hydrocolloids at the same time can be used as encapsulation vehicle due to their low viscosity at high solid content and good solubility (Silva, Vieira, & Hubinger, 2014). Gum Arabic (GA) is a well‐known hydrocolloid which has been substantially used in encapsulation process due its high water solubility, low viscosity, and formation of stable emulsion. GA is also believed to possess protective action in colloidal systems (Shaddel et al., 2018). Besides, GA is compatible with most macromolecular compounds such as gums, starches, carbohydrates, and proteins. However, due to its high cost and limited supply, GA alone cannot be applied in the colloidal systems. Therefore, maltodextrin (MD) and modified starches were used as alternative carrier materials (Krishnan, Bhosale, & Singhal, 2005). MD provides advantages such as low viscosity, good oxidative stability, and low cost; but it exhibits poor emulsifying capacity (Carneiro, Tonon, Grosso, & Hubinger, 2013). Hence, the mixture of GA, MD, and modified starch was reported more effective if compared with their application alone (Przybysz, Onacik‐Gür, Majtczak, & Dłużewska, 2016).

Based on above discussion, we are aimed first to extract and purify the fucoxanthin from *Sargassum angustifolium* that was followed by its encapsulation using PS. Then, for improving the fucoxanthin stability, PS encapsulating fucoxanthin was incorporated within the GA, MD and their complex (GA + MD) as the coating agent(s). The encapsulation efficiency and stability of fucoxanthin (light and temperature) during the storage period were examined. Additionally, a kinetic model was proposed to calculate thermal degradation parameters of fucoxanthin.

## 2 MATERIAL AND METHODS

### 2.1 Materials

Corn starch was purchased from Biological Processing Company. Ethanol (96% v/v), acetone, and n‐hexane were supplied from Dr. Mojallali Chemical Laboratories. *S. angustifolium* was purchased from Algae Resource Development Technology Company. The fucoxanthin standard (purity of >98%) was prepared from J&K Scientific Ltd. Silica gel was obtained from Nanochemia Company. Gum Arabic (GA), HPLC grade methanol, and acetonitrile were all obtained from Merck Co. Maltodextrin (MD) was obtained from Hangzhou Dingyan Chem Co.

### 2.2 Extraction and purification of fucoxanthin

Fucoxanthin was extracted from *S*. *angustifolium* dispersed in 90% ethanol (1:20 w/v) according to Kim et al. (2012) with a slight modification. Then, the extracted fucoxanthin product was purified using silica gel column chromatography (particle size 100–200 mesh) using hexane–acetone (6:4; v/v) as the mobile phase. To achieve a high purity product, the prepared fucoxanthin was further eluted by acetone.

### 2.3 Characterization of fucoxanthin

#### 2.3.1 High‐performance liquid chromatography (HPLC) analysis of fucoxanthin

The purity of fucoxanthin was confirmed using a HPLC system (KNAUER) equipped with an UV/Vis detector 2,600 and C18 column (sphere‐image, ODS‐2, 300× 4 mm; 5 µm). After passing fucoxanthin sample solution through a 0.45 μm membrane filter, an aliquot of 20 μl was injected into HPLC column. The mobile phase consisting of methanol/acetonitrile (50:50 v/v) eluted the column with a flow rate of 0.6 ml/min. Fucoxanthin was detected at wavelength of 450 nm, and area under the curve was compared to that of standard fucoxanthin (Norra, Aminah, Suri, & Zaidi, 2017).

#### 2.3.2 Nuclear magnetic resonance (NMR)

Molecular structure of the fucoxanthin derived from *S. angustifolium* was elucidated using ^1^H‐NMR and ^13^C‐NMR spectroscopy (Avance III 400 MHz, Bruker). Dimethyl sulfoxide‐d_6_ (DMSO‐d_6_) was used as the solvent.

### 2.4 Preparation of porous starch (PS)

Porous starch was prepared through heating an aqueous slurry of corn starch (5% w/v) at 90°C for 30 min as previously described (Oliyaei et al., 2019). The starch paste was cooled in refrigerator (5°C) for 48 hr to facilitate gelation process. The gel was then cut into several cylinder‐shaped pieces (1 × 1 cm) and kept at freezing temperature (−10°C) overnight. The frozen gels were subjected to the solvent exchange by 100% ethanol. The alcogels were equilibrated and stored in ethanol at room temperature three times for about 1 hr each time. Finally, the products were freeze‐dried.

### 2.5 Encapsulation of fucoxanthin

Encapsulation of purified fucoxanthin was carried out according to the method described by Wu et al. (2011) with slight modifications. The PS prepared in Section 2.4 was mixed with a 1:4 (w/v) volume of purified fucoxanthin (equivalent to ~0.58 mg fucoxanthin) and shaken (Incubator Shaker, JAL. TAJHIZ) in the dark for 24 hr. Finally, the whole mixture was dried by a vacuum dryer.

### 2.6 Modification of fucoxanthin‐loaded PS

Fucoxanthin‐loaded PS powders were further modified using different coating materials (GA, MD, and combined MD + GA in equal weight ratio) following the protocol by Wang, Ye, et al. (2012) with slight modifications. Fucoxanthin‐loaded PS without separating free fucoxanthin was incorporated in three different mass ratios with respect to the coating materials (1/3, 1/5, and 1/7 w/w) as shown in Table 1. Then, the mixtures were dispersed in distilled water (60°C) and homogenized with an Ultra‐Turrax homogenizer (IKA T18) at 715 *g* for 3 min. Finally, the samples were subsequently turned to powder using a freeze dryer.

**Table 1:** Composition of double encapsulated systems composed of fucoxanthin‐loaded PS and different coating materials

Mass ratio of fucoxanthin‐loaded PS: coating material (w/w)	Fucoxanthin‐loaded PS	Gum Arabic (GA)	Maltodextrin (MD)	GA + MD (1:1 w/w)
1/3	*	*	–	–
	*	–	*	–
	*	–	–	*
1/5	*	*	–	–
	*	–	*	–
	*	–	–	*
1/7	*	*	–	–
	*	–	*	–
	*	–	–	*

### 2.7 Encapsulation efficiency

The encapsulation efficiency was calculated from the mass ratio between content of fucoxanthin in PS and total amount of fucoxanthin used according to Equation 1:

(1) $$\text{Encapsulation}\;\;\text{efficiency}\;\left(\%\right)=M_{a}/M_{t}\times 100$$

where *M*_a_ is the content of fucoxanthin in PS, and *M*_t_ is the total amount of fucoxanthin added (Ravi & Baskaran, 2015).

### 2.8 Fourier‐transform infrared spectroscopy

Fourier‐transform infrared spectroscopy was performed to analyze functional groups and to provide an insight into structural characteristics of the modified fucoxanthin‐loaded PS. The spectra were recorded on a FTIR spectrophotometer (Tensor II, Bruker) in the wave number ranged from 4,000–500 cm^−1^.

### 2.9 Storage stability

The modified fucoxanthin‐loaded PS samples were investigated for their ability to preserve fucoxanthin at different environmental conditions. For this purpose, they were kept individually at 50, 4°C, under light and dark conditions for a total period of 4 weeks. The aliquots were collected in every 7‐day intervals for analysis of total fucoxanthin content at each condition, as follows (Equation 2):

(2) $$\eta \%=M_{t}/M_{a}\times 100$$

where *M*_t_ is the amount of fucoxanthin remaining in carriers at time *t*, and *M*_a_ is the amount of fucoxanthin which was initially encapsulated as similarly reported (Wang, Ye, et al., 2012).

### 2.10 Kinetic modeling of fucoxanthin degradation

Thermal degradation of the modified fucoxanthin‐loaded PS samples stored at 50°C was analyzed using zero‐order, first‐order and second‐order kinetic models according to Equations (3), (4), (5), respectively:

(3) $$C-C_{0}=-\text{kt}$$

(4) $$\ln\;\;C-\ln\;C_{0}=-\text{kt}$$

(5) $$1/C{\;\text{- 1/C}}_{0}=-\text{kt}$$

where C_0_ is the initial, *C* the final concentration of fucoxanthin, *t* the heating time and *k* the reaction rate constant.

### 2.11 Statistical analysis

Data were reported as mean ± standard deviation for triplicate determinations that were analyzed using analysis of variance (ANOVA) and Duncan's multiple range test at *p* < .05 by SAS software (SAS Institute).

## 3 RESULTS AND DISCUSSION

### 3.1 Preparation and characterization of fucoxanthin

**Figure 1:**
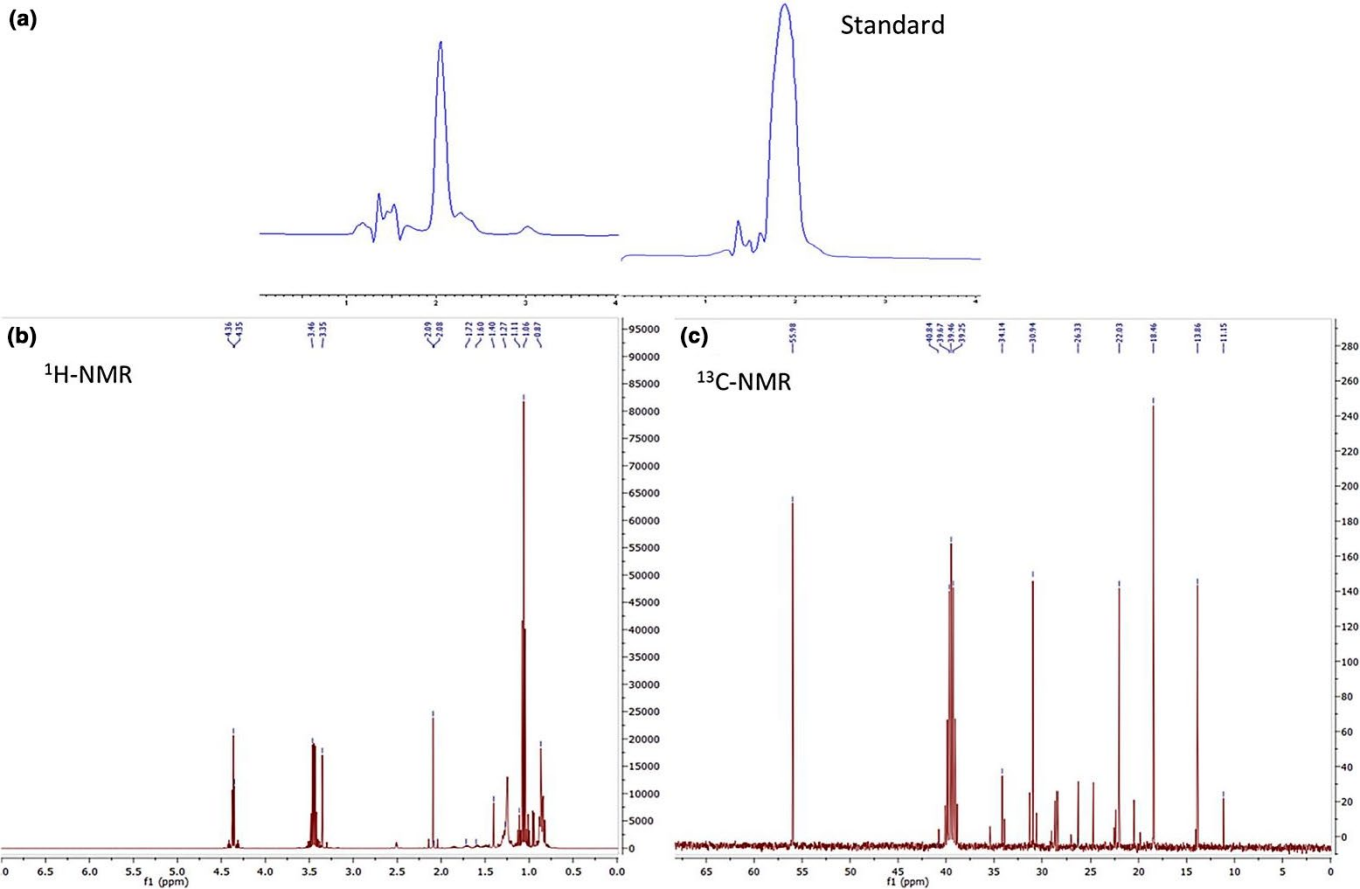
(a) HPLC chromatogram of fucoxanthin extracted from *Sargassum angustifolium*, (b) ^1^H‐NMR, and (c) ^13^C‐NMR spectra

The purified fucoxanthin was analyzed with HPLC, ^1^H‐NMR, and ^13^C‐NMR. The HPLC chromatograms of extracted and standard fucoxanthin showed one major peak came out at the retention time of about 2 min (Figure 1a). The minor peaks in purified fucoxanthin can be related to the *cis‐isomers* of fucoxanthin as suggested by other relevant studies (Yip, Joe, Mustapha, Maskat, & Said, 2014; Zailanie & Purnomo, 2017). Molecular structure of fucoxanthin has many conjugated double bonds. These double bonds can interchangeably transfer from trans‐isomers to *cis‐isomers* under different environmental conditions (heat, light or oxygen). This is known as the first degradation step of fucoxanthin (Piovan, Seraglia, Bresin, Caniato, & Filippini, 2013). Moreover, the formation of apocarotenoids, low molecular weight compounds resulting in breakdown of the C = C bonds in the carotenoid structure, may be observed (Cordenonsi et al., 2017). In addition, the recovered fucoxanthin exhibited the purity about 54% based on the HPLC data.

^1^H‐NMR and ^13^C‐NMR spectra of the purified fucoxanthin were shown in Figure 1b,c, respectively. The functional groups of fucoxanthin were identified after assigning peaks with different chemical shifts in these spectra. The high resolution peaks were identified with the functional groups in the molecule. The ^13^C‐NMR revealed six aliphatic CH_3_, two cyclohexane, three CH_2_ cyclohexane, and a CH_2_. ^1^H‐NMR results showed methyl singlet form (16‐H, 17‐H, 16’‐H, 17’‐H, 18’‐H), methyl in double bond (CH3‐C=) (20‐H, 19’‐H, 20’‐H), hydroxyl methyl (3’‐H), ten protons of a double bond (H‐C=), methylene (2’‐H, 4’‐H). The ^1^H‐NMR and ^13^C‐NMR results were almost identical to that of fucoxanthin described elsewhere (Kim et al., 2012; Zailanie & Purnomo, 2017).

### 3.2 Encapsulation efficiency of fucoxanthin

**Figure 2:**
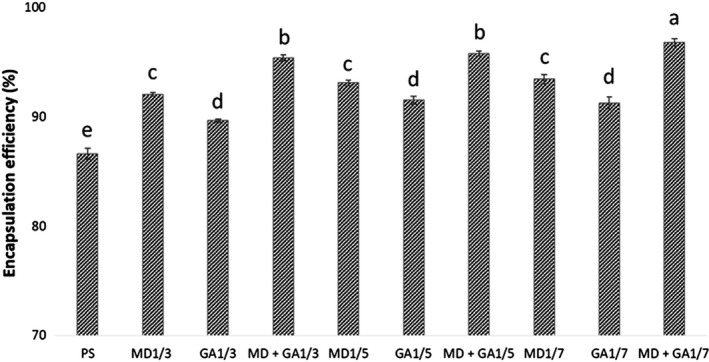
Encapsulation efficiency (%) of fucoxanthin‐loaded PS samples alone or after coating by maltodextrin (MD), gum Arabic (GA), and their combination (1:1 w/w MD: GA) in different weight ratios (1/3, 1/5, and 1/7 w/w)

Efficiency of the different coating materials, MD, GA, or their combination (MD + GA), in encapsulating the fucoxanthin‐loaded PS was calculated and compared. Figure 2 presents the percentage of fucoxanthin finally retained within each matrix. The results revealed a significant enhancement in encapsulating the fucoxanthin‐loaded PS occurred by changing the coating materials. The encapsulation efficiency of fucoxanthin in PS was found to be relatively high (86.6%), which was further increased by sequential modification with gum‐based materials. GA alone resulted in the lowest amount of encapsulated fucoxanthin regardless of the ratio. However, the encapsulation efficiency was found relatively higher in MD in comparison to GA. It should be noticed that fucoxanthin was encapsulated first with PS (without washing and removing the surface fucoxanthin) and then mixed with gum(s). Meanwhile, PS presented high encapsulation efficiency because of its “sponge‐like” structure with opening holes which extending from the surface to the center and allows molecules to enter its structure which causes large specific surface area and high efficiency (Belingheri et al., 2015; Glenn et al., 2010; Zhang et al., 2012). The porous structure of PS was observed in our previous study (Oliyaei et al., 2019). Moreover, the high adsorption capacity and encapsulation efficiency of fucoxanthin‐loaded PS was obtained in our previous study (Oliyaei, Moosavi‐Nasab, Tamaddon, & Fazaeli, 2020). Indeed, fucoxanthin entered in holes and filled them; however, partially of fucoxanthin was adsorbed on the surface of PS (Oliyaei et al., 2020).

de Barros Fernandes, Borges, and Botrel (2014) obtained similar results for the encapsulation of rosemary essential oil using the combined modified starch and MD. As another example, Ballesteros, Ramirez, Orrego, Teixeira, and Mussatto (2017) demonstrated a similar observation in encapsulation process of coffee phenolic compounds. These authors reported high encapsulation efficiency using MD. The mass ratio of different coating materials was also considered as an important factor that may cause improvement in the encapsulation efficiency. It was found that 92.04%, 93.09%, and 93.43% of fucoxanthin was retained for the samples coated with 1/3, 1/5, and 1/7 w/w of MD, respectively. However, the differences were not determined significant (*p* > .05). Indeed, fucoxanthin‐loaded PS was incorporated into MD, GA and MD + GA and gums which act as second barrier. Thus, fucoxanthin should be expelled from PS first to get released from PS coated by gum (s) into medium. Thus, it seems that the double encapsulation and higher mass ratio caused more protective effect than PS alone.

Even though, MD causes remarkably high encapsulation efficiency, it appears that its effect may not be enough for several food applications. Thus, to increase its effect to the required levels, the mixture of MD with GA is proposed for retaining of fucoxanthin inside PS and improving the encapsulation efficiency. This is known that GA is a suitable encapsulation agent because of its emulsifying capacity (de Barros Fernandes et al., 2014).

Interestingly, the combination of MD and GA exhibited better encapsulation efficiency of fucoxanthin‐loaded PS, which resulted in the highest encapsulation efficiency of 96.79% using MD + GA (1/7 w/w). Based on these results, the combination of GA and MD is suggested for the double encapsulation process because their combination can provide the highest encapsulation and consequently a good barrier function in preserving fucoxanthin entrapped in PS matrix. These results were in close agreement with those reported by Y. Wang, Ye, et al. (2012), which showed the highest efficiency of 94.4% if lutein was microencapsulated in mixture of PS and gelatin. High encapsulation efficiency may be explained by the sponge‐like structure of PS, high water absorption, and good adhesive properties. Wang, Shao, Wang, and Lu (2012) reported that PS combined with β‐cyclodextrin can improve the encapsulation efficiency of allicin. Moreover, Wang, Lu, Lv, and Bie (2009) achieved approximately 91.6% encapsulation efficiency when curcumin was encapsulated inside the mixture of PS and gelatin. In another report, Sun et al. (2018) presented the high encapsulation efficiency of fucoxanthin encapsulated by MD (92.35%) and GA (86.48%) alone. Based on these findings, it appears that the combined MD and GA are more appropriate to meet the requirement of food industries.

### 3.3 FTIR spectroscopy

**Figure 3:**
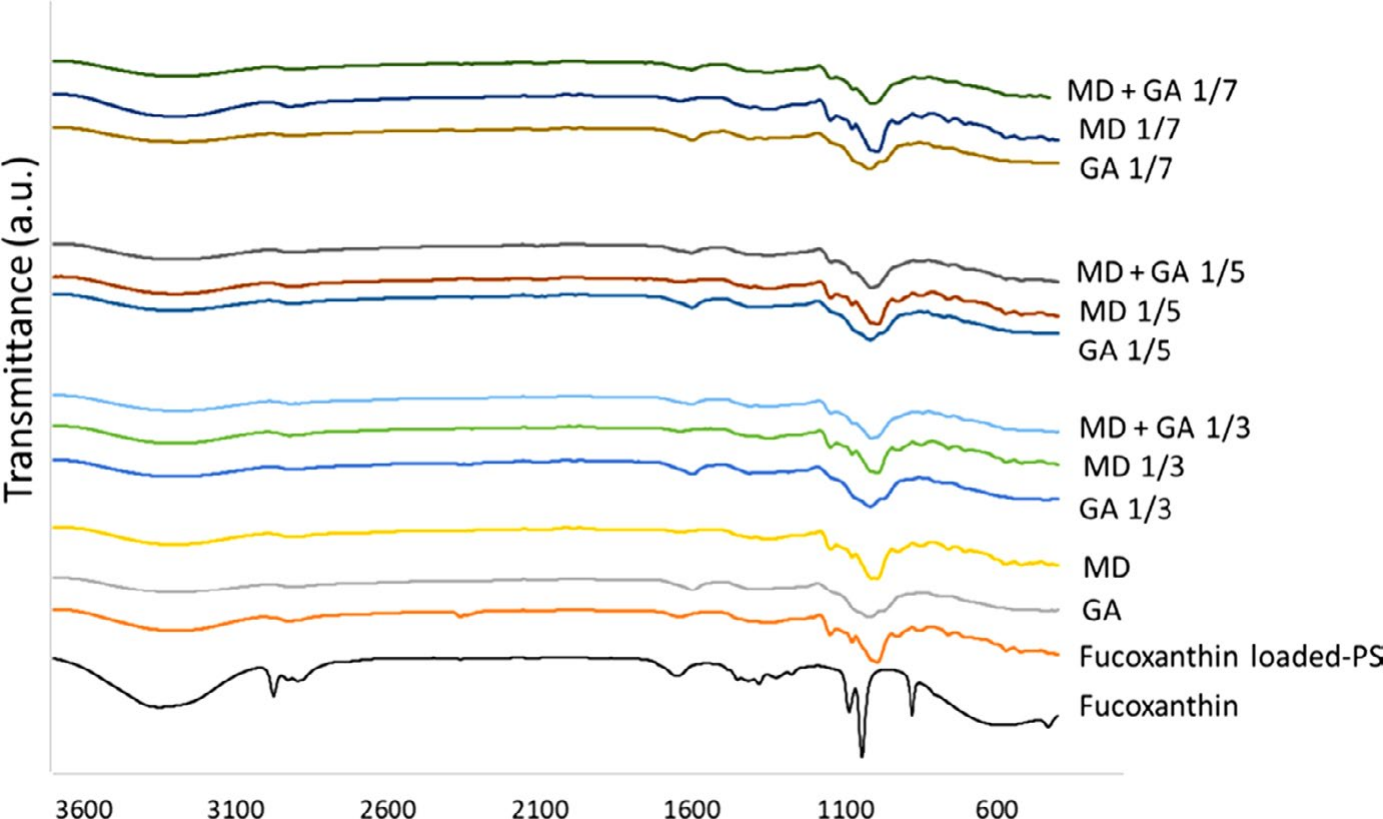
FTIR spectra of the extracted fucoxanthin, the fucoxanthin‐loaded PS alone or after coating by maltodextrin (MD), gum Arabic (GA), and their combination (1:1 w/w MD:GA) in different weight ratios (1/3, 1/5, and 1/7 w/w)

Fourier‐transform infrared analysis was performed to investigate the structural changes of fucoxanthin in the encapsulation process. Figure 3 shows the FTIR spectra of fucoxanthin, fucoxanthin‐loaded PS, and the loaded PS samples which were further coated by GA, MD, and their combination at different weight ratios. In the case of fucoxanthin‐loaded PS, the characteristic peak at 3,346 cm^−1^, which was assigned to stretching vibration of hydroxyl groups of either starch or fucoxanthin, shifted to the lower wavenumbers of 3,299 cm^−1^, indicating possible interactions between these two compounds. In addition, small peaks observed around 994 and 759 cm^−1^ can be ascribed to the interaction of –C = C‐ fucoxanthin with C‐O of PS. FTIR spectra of GA similarly showed main characteristic bands at 1,019 cm^−1^, 1,599 cm^−1^, and 3,287 cm^−1^, which are assigned to the stretching vibration of C‐O, C = O, and O‐H groups, respectively (Leonor et al. (2013). FTIR spectra of MD were also characterized by two bands located at 1,357 and 846 cm^−1^, which are generally associated with to polysaccharide structure. Furthermore, two additional bands observed at 1,076 and 1,147 cm^−1^ can be assigned to C‐O stretching vibration in MD. The characteristic bands detected in GA and MD were found to be like those observed by Dewi, Kurniasih, and Purnamayanti (2017). The FTIR spectra of noncoated fucoxanthin‐loaded PS were compared with the fucoxanthin‐loaded PS coated by GA, MD, and MD + GA. The FTIR spectra of fucoxanthin‐loaded PS coated with GA showed only slight changes. The peak located at 2,360 cm^−1^ which is assigned to C‐H stretching vibration in fucoxanthin‐loaded PS almost disappeared when GA was used in higher ratios (1/5 and 1/7). Besides, the band at 3,299 cm^−1^ slightly shifted to 3,284 cm^−1^. These observations confirmed possible interactions between GA and the fucoxanthin‐loaded PS. In addition, the interaction between fucoxanthin‐loaded PS and MD was evidenced by the disappearance of the band at 2,260 cm^−1^ in of the fucoxanthin‐loaded PS and emergence of a new shoulder peak at 2,112 cm^−1^, which was more evident in the high ratio of MD. The absorption band about 1,358 cm^−1^ revealed also the presence of MD in the sample. The similar patterns were observed in the FTIR spectra of MD‐ or MD + GA‐coated samples, indicating the physical interaction between the fucoxanthin‐loaded PS and the coating materials.

### 3.4 Storage stability of encapsulated fucoxanthin

**Figure 4:**
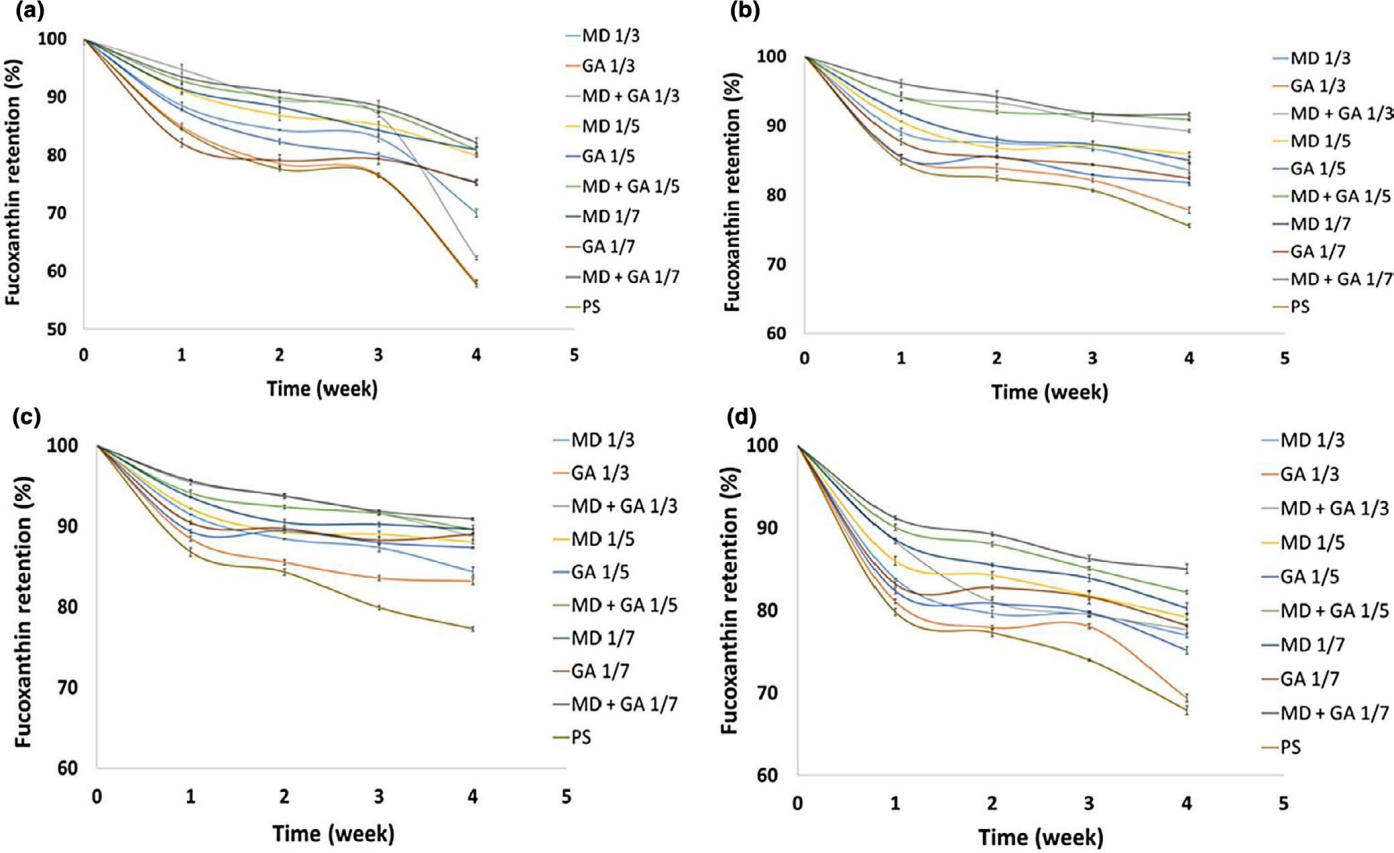
Fucoxanthin retention under different environmental conditions, light (a), dark (b), 4°C (c) and 50°C (d) for a duration of four weeks; fucoxanthin‐loaded PS alone (PS) or after coating by maltodextrin (MD), gum Arabic (GA), and their combination (1:1 w/w MD + GA) in different weight ratios (1/3, 1/5, and 1/7 w/w)

High temperature or high intensity light exposure can cause detrimental changes to the structure of fucoxanthin molecule due to the high levels of sensitivity of fucoxanthin to elevated temperature and light (Indrawati, Sukowijoyo, Wijayanti, & Limantara, 2015). Figure 4 shows the retained percentage of fucoxanthin in different samples following exposure to different conditions (lightness, darkness, 4 and 50°C) for a duration of 4 weeks. The fucoxanthin retention was greatly influenced by the presence of light so that it decreased from 84.50% to 57.73% when it was encapsulated with PS only while it changed from 84.9% to 58.2% in when fucoxanthin‐load PS was modified with GA (1/3 w/w). Pigment structure can be strongly affected by light exposure as reported by other authors (Gandía‐Herrero, Jiménez‐Atiénzar, Cabanes, García‐Carmona, & Escribano, 2010; Zuanon, Malacrida, & Telis, 2013). This enhanced retention of fucoxanthin was also noted in the other samples stored under the same light exposure. Interestingly, the combination of MD + GA, particularly when used at high ratio (1/7 w/w), provided a better barrier in protecting the fucoxanthin that led to the retention of 82% fucoxanthin at the end of the storage period. Comparing individual GA and MD coating, those fucoxanthin‐loaded PS samples coated with MD showed higher fucoxanthin retention than those with GA coating. In a recent study, Zhao et al. (2019) evaluated the kinetics of fucoxanthin degradation and revealed that light can accelerate the degradation of *trans* form of fucoxanthin via photodegradation and photoisomerization reactions. On the contrary, the dark conditions demonstrated the superior inhibitory effect on the fucoxanthin degradation. Retention percentages of fucoxanthin‐loaded PS after the modification with MD + GA reached to 89.3%, 90.9%, and 91.6% after 4 weeks of storage when different ratios of 1/3, 1/5, and 1/7 w/w were employed, respectively. However, under the same conditions, the retention of fucoxanthin‐loaded PS was observed to be still low at the end of the storage (75.5%).

Apart from the light exposure, it was found that for all modified samples, fucoxanthin retention (%) exhibited significantly high values when they were kept at 4°C, in comparison to those stored at 50°C. The retention values of fucoxanthin encapsulated in PS alone were calculated 77.26% and 67.9% at 4 and 50°C, respectively. These results are consistent with the similar report on thermal degradation and isomerization of fucoxanthin at 60°C (Zhao et al., 2019). Considering the possible impact of the coating process on thermal degradation of fucoxanthin, the highest stability was remarkably obtained for the fucoxanthin‐loaded samples when MD + GD in the ratio of 1/7 w/w was used (90.9%), which showed only a modest drop to 85.0% after exposure to 50°C. On the other hand, the results of fucoxanthin stability at 50°C revealed that there was no significant difference between fucoxanthin retention of the GA or MD coated samples (especially in ratio of 1/3 w/w) after second and third weeks of storage. However, significant changes in fucoxanthin retention occurred in higher modification ratios (*p* < .05). In corroboration with previous findings on encapsulation efficiencies, it was revealed the encapsulated fucoxanthin can be retained more if high ratio of desired gum (either GA or MD) is used. Besides, it was demonstrated that the combinatorial effect of two gums (GA and MD) offers a great protection over the encapsulated fucoxanthin. These coating materials are assumed to act as physical barrier against the penetration of oxygen, light, or heat to ultimately protect the encapsulated material (Chranioti, Nikoloudaki, & Tzia, 2015). Wang, Ye, et al. (2012) found that lutein encapsulated in PS and gelatin mixture showed high stability after light exposure over 30 days in which the loss of encapsulated lutein was only 10.5%. In addition, the authors found less than 1% reduction of encapsulated lutein when stored at an elevated temperature ranging from 0 to 100°C for 1 hr. Similar results were reported on the light and temperature stability of curcumin after being encapsulated in PS and gelatin mixture (Wang et al., 2009). This further confirms the critical role of encapsulating agents on the storage stability of sensitive ingredients. Wang, Ye, et al. (2012) investigated also the effect of light on stability of allicin encapsulated with PS and β‐cyclodextrin. They found a total loss of 33.5% and 13.4% when allicin stored in free and the encapsulated form for 30 days, respectively. Moreover, the authors verified that the encapsulated allicin using PS and β‐cyclodextrin was stable at temperatures below 60°C, in which they exhibited a slow reduction of about 13.3% up to 100°C.

### 3.5 Modeling the kinetics of fucoxanthin degradation

**Figure 5:**
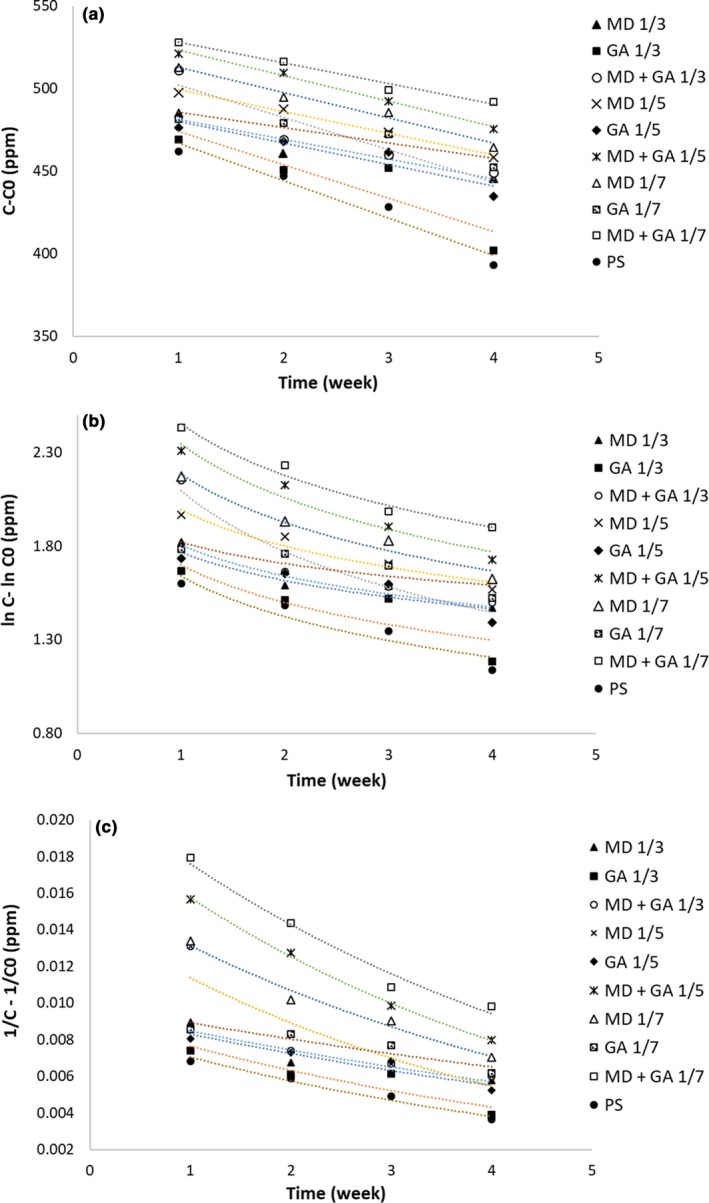
Zero‐ (a), first‐ (b) and second‐ (c) order kinetic thermal degradation of fucoxanthin‐loaded PS alone or after coating by maltodextrin (MD), gum Arabic (GA), and their combination (1:1 w/w MD + GA) in different weight ratios (1/3, 1/5, and 1/7 w/w)

Kinetics of fucoxanthin thermal degradation was quantitatively explained by choosing any of zero‐, first‐ or second‐order equation at temperature of 50°C. With an increase in the storage period, the concentration of fucoxanthin gradually dropped and fucoxanthin undergo a degradation process (Figure 5). Table 2 summarizes the thermal degradation parameters (K and *R* ^2^) of the fucoxanthin‐loaded PS and after being modified by the coating materials. High values of the regression coefficients (*R* ^2^) were similarly determined, indicating good fitness of data to the all proposed kinetic models. Nevertheless, it seems that the first‐order kinetic is more appropriate for describing the thermal degradation of fucoxanthin (Table 2). Other studies on this topic also came up to the same conclusion as the first‐order kinetics can explain the thermal degradation of carotenoid‐based compounds (Achir, Randrianatoandro, Bohuon, Laffargue, & Avallone, 2010; Lemmens et al., 2010). For instance, Xiao et al. (2018) evaluated thermal degradation of some carotenoids such as lutein, zeaxanthin, β‐cryptoxanthin, and β‐carotene at 25, 35, and 45°C. They confirmed that the degradation of carotenoids was best described by first‐order kinetic model. Although, on contrary to our findings, Sun et al. (2018) found the second‐order kinetic model for describing thermal degradation of fucoxanthin microencapsulated by various coating agents.

**Table 2:** Kinetic parameters for the thermal degradation of fucoxanthin‐loaded PS alone or after by maltodextrin (MD), gum Arabic (GA), and their combination (1:1 w/w MD + GA) in different weight ratios (1/3, 1/5, and 1/7 w/w)

Samples	Zero‐order kinetic		First‐order kinetic		Second‐order kinetic	
	K	*R* ^2^	K	*R* ^2^	K	*R* ^2^
PS	108.20 ± 7.45	.96	0.35 ± 0.05	.89	0.001 ± 0.00	.97
MD 1/3	115.77 ± 4.11	.88	0.40 ± 0.04	.93	0.002 ± 0.00	.86
GA 1/3	110.91 ± 7.29	.80	0.37 ± 0.05	.73	0.001 ± 0.00	.82
MD + GA 1/3	118.15 ± 6.79	.86	0.43 ± 0.07	.93	0.002 ± 0.00	.82
MD 1/5	119.87 ± 4.29	.99	0.44 ± 0.04	.94	0.002 ± 0.00	.82
GA 1/5	115.11 ± 4.50	.89	0.40 ± 0.04	.80	0.002 ± 0.00	.91
MD + GA 1/5	124.99 ± 4.98	.99	0.50 ± 0.06	.96	0.003 ± 0.00	.99
MD 1/7	122.42 ± 5.00	.98	0.47 ± 0.06	.97	0.002 ± 0.00	.98
GA 1/7	117.89 ± 3.30	.84	0.42 ± 0.03	.97	0.002 ± 0.00	.86
MD + GA 1/7	127.30 ± 4.07	.98	0.53 ± 0.06	.98	0.003 ± 0.00	.97

## 4 CONCLUSIONS

The present study provides a double encapsulation process for modifying the fucoxanthin‐loaded PS with different coating materials in terms of the encapsulation efficiency and stability constant in different environmental conditions. The data revealed that the stability of PS‐encapsulated fucoxanthin was greatly influenced by the change in temperature or the presence/absence of light exposure. High temperature along with prolonged storage caused a remarkable reduction in amount of the entrapped fucoxanthin. Interestingly, the modification of fucoxanthin‐loaded PS with different gum materials not only improved the encapsulation efficiency, but also it allowed an enhancement in stability of the PS‐encapsulated fucoxanthin when exposed to light and high temperature for 4 weeks storage. In addition, MD coating showed better performance in comparison with GA; however, their combination (MD + GA), especially in high ratio, was found to be more suitable to avoid the immediate loss of fucoxanthin during harsh environmental conditions. To predict the thermal degradation of encapsulated fucoxanthin, the first‐order kinetics model was fitted, explaining nonlinear thermal degradation of the encapsulated fucoxanthin. Taking altogether, the modification of encapsulated fucoxanthin‐loaded PS with MD and GA as the coating agents demonstrated excellent encapsulation efficiency and preservation of fucoxanthin against heat and light exposure. Therefore, the double encapsulation of fucoxanthin can be useful for those industries which are required to protect their sensitive ingredients from severe environmental conditions.

## CONFLICT OF INTEREST

We declare that we have no conflict of interest.

## ETHICAL APPROVAL

This study does not involve any human or animal testing.
